# A structured framework to improve usability in EHR implementation: a user-centered case study in Brazilian mental healthcare

**DOI:** 10.3389/fdgth.2025.1676631

**Published:** 2026-01-05

**Authors:** Fernanda Peron Gaspary, Daniel Baia Amaral, Cristian Vinicius Fagundes, Luis Felipe Dias Lopes, João Francisco Pollo Gaspary

**Affiliations:** 1 Franciscan University, Santa Maria, Brazil; 2 Institute AuBento—Center for Education, Clinical Practice, and Research in Orthomolecular and Integrative Medicine, Santa Maria, Brazil; 3 Center for Social and Human Sciences, Postgraduate Program in Administration, Federal University of Santa Maria, Santa Maria, Brazil

**Keywords:** digital health innovation, electronic health records (EHR), human-centered design, implementation science, interoperability, mental healthcare, service design, usability

## Abstract

**Background:**

Electronic Health Record (EHR) systems are central to digital health transformation, yet usability challenges continue to constrain their effectiveness, particularly in mental healthcare contexts.

**Objectives:**

To develop and describe a structured, user-centered framework for improving EHR usability based on a Brazilian outpatient mental health case study.

**Methods:**

This qualitative design research study, guided by the Double Diamond design methodology, followed four iterative phases (Discover, Define, Develop, Deliver) and conducted qualitative interviews with 21 healthcare professionals. Data were organized using the Certainties, Suppositions, and Doubts (CSD) matrix and triangulated through heuristic evaluation and prototype testing.

**Results:**

Key barriers included non-standardized navigation flows, limited integration with external systems, and inflexible documentation structures. Based on these findings, the study proposes design-driven improvements such as customizable templates, real-time validation features, and workflow-specific interface adjustments.

**Conclusions:**

By integrating service design logic with usability-driven interface adaptations and addressing both systemic usability gaps and contextual demands, this research contributes actionable insights for advancing human-centered EHR innovation, with particular relevance to complex mental healthcare workflows.

## 1 Introduction

The digital transformation of healthcare has initiated a paradigmatic shift in how medical data and services are managed, improving access, coordination, and continuity of care (1–4). This shift increasingly demands that Electronic Health Record (EHR) systems not only function as digital repositories but also serve as interoperable, adaptive infrastructures capable of supporting complex clinical and administrative workflows across varied healthcare contexts. Among the most significant innovations of this transition are cloud-based EHR platforms, which enable secure, remote storage and real-time sharing of clinical information across providers and institutions (4–6). By eliminating physical storage requirements and reducing service delays and errors, such systems enhance clinical decision-making and patient safety (6).

In Brazil, the widespread adoption of EHRs in outpatient clinics and private practices has been accompanied by the integration of telemedicine, appointment scheduling, and financial management tools into unified platforms (7), although dissemination and scalability still face important challenges (8). However, the effectiveness and reliability of these digital systems depend not only on the sophistication of their underlying technologies but also on critical attributes such as usability, interoperability, and compliance with regulatory standards. These attributes are central to ensuring that EHR implementation translates into real-world utility—particularly in resource-constrained environments, where platforms must evolve incrementally to accommodate diverse clinical needs. Healthcare professionals and patients alike face barriers when interfaces are unintuitive or poorly integrated with external systems, potentially limiting the adoption and utility of these platforms (7).

Data privacy and security represent another major challenge for digital health tools. Patient data are classified as sensitive and demand advanced protection mechanisms (9, 10). In Brazil, the General Data Protection Law imposes strict provisions on the collection, storage, and sharing of personal health information, compelling digital platforms to adopt robust encryption, access control, and compliance protocols (11, 12). Ensuring privacy is not only a legal obligation but also a cornerstone for fostering trust in digital health ecosystems. As digital health platforms expand, maintaining compliance while supporting innovation remains a delicate balance—particularly when integrating patient-facing functionalities and third-party data flows.

Among the leading cloud-based platforms in the Brazilian market, Afya iClinic has emerged as a prominent choice for clinics and individual practitioners (13). The system integrates multiple functionalities—EHR, appointment scheduling, telemedicine, financial management, and medical marketing—within a single ecosystem. While designed to streamline clinical and administrative workflows and enhance patient access, the platform must be continually reassessed to ensure it meets the evolving demands of the digital health landscape and delivers a high-quality user experience (13).

This study undertakes a critical evaluation of the Afya iClinic platform (2025), with a specific focus on identifying its strengths and shortcomings from a usability, security, and interoperability perspective—key pillars that directly influence the success of EHR implementation and adoption at scale. Drawing on user feedback from healthcare professionals—including physicians and administrative staff—the research aims to propose innovative, evidence-based design improvements aligned with user needs and clinical workflows.

To reinforce the practical scope of this evaluation, the research integrates service design tools such as user journey mapping, service blueprints, and low-fidelity prototyping. These methods enabled the translation of insights into actionable improvements, providing visual and functional representations of the proposed changes. This generative approach supports incremental innovation and promotes adaptability across EHR systems facing similar contextual constraints, aligning with value-based healthcare frameworks that highlight the strategic relevance of intangible assets as drivers of organizational value creation (14) and increase organizational resilience (15).

Although Afya iClinic (13) is well-established and widely implemented in Brazil, it still presents usability challenges that may hinder its optimal performance. Key concerns include complex interface structures, limited system integration capabilities, and data security vulnerabilities (16). These factors can compromise user satisfaction and deter full engagement with the platform's functionalities—ultimately limiting the systemic value of EHR implementation efforts. Therefore, the guiding research question of this investigation is: What areas of the Afya iClinic platform require improvement, and how can its design be enhanced to optimize usability, efficiency, and data security? In addressing this question, the study aims to identify critical pain points and recommend design-driven interventions that align with user expectations and emerging best practices in health informatics.

The rationale for selecting Afya iClinic as the subject of this study lies in its high adoption rate among Brazilian healthcare providers and its comprehensive service offerings (17). Enhancements proposed in this research have the potential to generate a meaningful impact, improving operational efficiency and user experience for a broad segment of the healthcare market. Moreover, the platform's integrated design—spanning clinical, administrative, and financial domains—makes it an ideal case study for analyzing complex digital health systems.

Another compelling justification for this analysis is Afya iClinic's technological maturity and emphasis on cloud-based architecture, which includes state-of-the-art security protocols such as data encryption and multi-factor authentication (17). These features position the platform as a leader in digital health infrastructure, yet ongoing usability evaluation remains critical. Evaluating user experience (UX) from a clinical and operational perspective is essential to identify improvement opportunities that ensure the platform's continued relevance and effectiveness (18, 19).

This study not only contributes to the specific optimization of the Afya iClinic platform but also offers broader implications for other digital health tools. By articulating a user-centered evaluation process supported by empirical data, the research delivers transferable insights and a replicable methodology for improving usability and functionality in similar platforms. Ultimately, the project aims to enhance operational efficiency, patient care quality, and digital adoption across the healthcare sector.

The primary objective of this research is to analyze the Afya iClinic platform and propose innovative solutions that improve its usability, security, and operational efficiency. Specific objectives include evaluating the user interface and experience, examining security features for LGPD compliance, assessing the efficiency of core functionalities (scheduling, EHR, telehealth, financial tools), and reviewing system interoperability with third-party solutions. By incorporating feedback from key stakeholders, the study provides a comprehensive, evidence-based analysis that supports targeted platform improvements and contributes to the advancement of digital health implementation in Brazil.

Given these considerations, this study adopts a user-centered design approach to assess and improve the usability of the Afya iClinic platform in mental health contexts. By integrating exploratory fieldwork, heuristic evaluation, and iterative design processes, the research aims to translate insights into design interventions that reflect the situated needs of mental health professionals, while contributing to the broader discourse on user-centered innovation in digital health systems. This focus aligns with recent calls to strengthen the human factors perspective in EHR implementation, particularly in mental health care settings where nuanced workflows and user sensitivities demand tailored digital solutions.

## 2 Methods

This study is designed as a single-case study focused on the Afya iClinic platform (2025), a widely used EHR system in Brazil, examined here as a real-world implementation case to explore usability improvement strategies within digital health systems. It adopted the Double Diamond design methodology (20–23) as the structural framework for analyzing and developing user-centered solutions.

### 2.1 Implementation of the double diamond model

**Figure 1:**
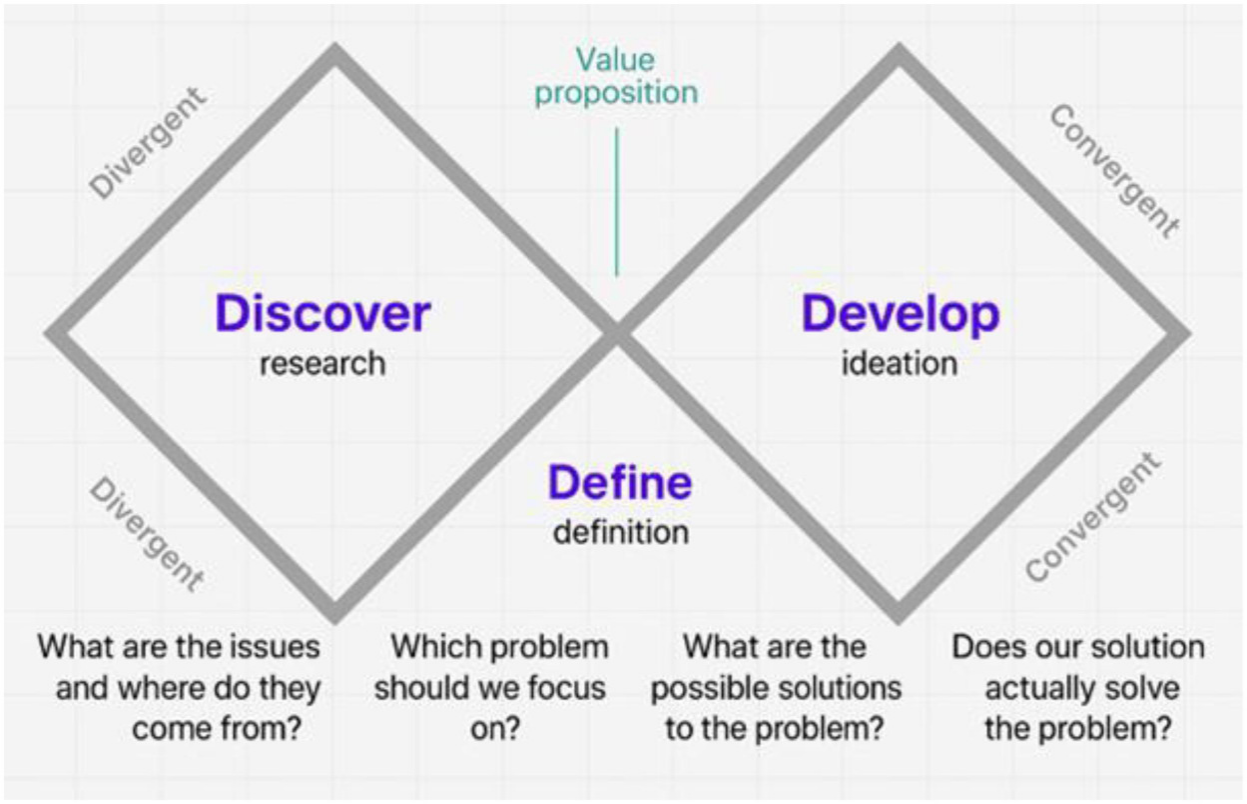
Application of the double diamond model.

Widely applied in service design and innovation, the Double Diamond model is composed of four sequential phases—Discover, Define, Develop, and Deliver—that alternate between divergent and convergent modes of thinking. This structure provided an appropriate methodological scaffold for evaluating the Afya iClinic platform, as it supports user-centered exploration, iterative refinement, and progressive consolidation of insights. In this study, the overall workflow followed the canonical representation of the model (Figure 1), ensuring alignment with internationally recognized design-science practices while preserving flexibility for context-specific adaptations.

During the Discover phase, the team conducted an exploratory investigation into the usage context of Afya iClinic. Semi-structured interviews (*n* = 21) were held with mental health professionals—including physicians, psychologists, and support staff—to identify usability challenges, integration gaps, and security concerns. Patient feedback was also gathered to evaluate the perceived quality of care provided through the platform. Concurrently, document analysis of Afya iClinic's user manuals and privacy policies was performed to assess the platform's alignment with the Brazilian General Data Protection Law (LGPD). A distinctive element of the study was the use of the Certainties, Suppositions, and Doubts (CSD) matrix (24), which structured the interview process and supported the synthesis of exploratory data. Certainties helped anchor the analysis, while suppositions and doubts guided further inquiry.

A design specialist on the research team conducted a heuristic evaluation of the Afya iClinic interface, identifying issues in usability, navigation, and integration. This qualitative assessment was essential in triangulating the feedback from interviews and ensuring the research focus remained consistent with user-identified challenges.

In the Define phase, the collected data were synthesized to identify recurring patterns and prioritize critical issues affecting user experience. Personas and user journey maps were developed to represent distinct user profiles and contextualize specific usability barriers. These personas were derived from participant segmentation and mapped across distinct interaction phases using service journey frameworks. Additionally, a service blueprint was developed to visualize the systemic relationships between user actions, backstage processes, and technological support across all stages of consultation. The CSD matrix was again utilized to validate assumptions and address unresolved issues from the initial interviews. For instance, the assumption that the Afya iClinic interface was intuitive was critically examined and contrasted with interview data, which revealed notable difficulties in navigation and task execution.

The central research problem that emerged from this phase was: How can the usability and system integration of Afya iClinic be improved to optimize the clinical and administrative workflow of healthcare professionals while enhancing the overall user experience?

In the Develop phase, brainstorming sessions were held with multidisciplinary participants—including healthcare professionals and design experts—to generate creative solutions for the identified challenges. The most promising ideas were translated into low-fidelity prototypes, including wireframes and mockups, which were then tested with focus groups composed of platform users. These sessions emphasized divergent thinking to ensure that a broad range of potential solutions was considered before narrowing down to the most viable ones.

The final phase, Deliver, involved testing, refining, and validating the proposed improvements. These prototypes included documentation flow options and interface layout proposals, aligned with pain points and usability opportunities derived from participant input and heuristic criteria. Screens were designed to reflect simplified, customizable workflows, with contextual guidance and automation features integrated as core design principles. Usability tests were conducted to assess whether the redesigned components met user expectations and improved platform efficiency. Based on feedback from these sessions, iterative adjustments were made to the prototypes to align with user needs and project goals. Key Performance Indicators (KPIs), as defined by HCI.CARE (25), were employed to measure the impact of the implemented solutions. These included user satisfaction, error reduction, and operational efficiency.

The CSD matrix was not only instrumental during the Discover phase, but also served as a transversal analytical scaffold throughout the study, guiding assumption synthesis and prioritization of solutions. For example, the certainty that digital records are generally preferred over paper ones prompted a deeper investigation into the aspects that make EHRs more valuable. Similarly, the assumption of intuitive usability was challenged and tested through interview feedback. Uncertainties about system interoperability with external tools guided specific questions and subsequent solution development.

### 2.2 Participant profile

The study aimed to capture diverse perspectives from healthcare professionals in mental health settings. Participants were selected based on gender, age, years of experience, and digital literacy to ensure representation across user profiles. To ensure consistency and depth in data collection, the research team employed a semi-structured interview script developed specifically for this study. The guide was designed to explore participants’ professional backgrounds, prior experience with EHR systems, patterns of Afya iClinic usage, and perceived opportunities for improvement. The structured yet flexible format allowed for in-depth insights while maintaining comparability across interviews. Table 1 summarizes the selection criteria.

**Table 1:** Participant characteristics.

Criteria	Categories	Rationale
Gender	Male/Female	Ensures inclusive analysis and highlights gender-based variations in user experience.
Age range	20–30/31–40/41–50/50+	Reflects generational differences in digital fluency and adaptability.
Professional experience	5 years/10 years/15+ years	Accounts for varying expectations and exposure to multiple digital platforms.
Technology familiarity	Digital Native/Digital Immigrant	Assesses perceived usability across different levels of digital proficiency.

Interview transcripts were guided by a standardized script covering usability, security, integration, and overall user experience. The sample included both digital natives (highly tech-savvy professionals) and digital immigrants (those with limited comfort using digital interfaces). Despite a limited interview pool compared to broader EHR studies, the sample of 21 professionals was sufficient to identify key usability trends and inform design priorities.

The integration of the Double Diamond model with the CSD matrix provided a structured, user-centered methodology grounded in empirical data. The iterative process ensured that solutions were both context-sensitive and validated in practice.

### 2.3 Literature review

To inform the theoretical grounding and methodological framing of this study, a narrative literature review was conducted across two major electronic databases—LILACS (26) and MEDLINE (27)—with a focus on publications from the past five years. The search strategy was guided by descriptors derived from the *Descritores em Ciências da Saúde (DeCS)* taxonomy, including: *usability in healthcare digital tools*, *user-centered interface*, *electronic medical records*, *General Data Protection Law (LGPD)*, *healthcare innovation*, and *design process improvement*. Additional manual searches were performed to identify grey literature and design-specific sources related to service blueprinting and heuristic evaluation in digital health systems.

Studies were selected based on their conceptual contribution to three core analytical domains: (i) barriers to EHR usability in outpatient care, (ii) regulatory and ethical considerations in platform design (e.g., LGPD), and (iii) best practices in applying user-centered methodologies to clinical software environments. Articles published in English or Portuguese were eligible, and both peer-reviewed and institutional sources were included to support methodological triangulation.

Although the review was not structured as a formal systematic synthesis, the inclusion criteria emphasized practical applicability to real-world design and implementation challenges in digital health. The selected studies contributed to the conceptual backbone of the research by shaping the interview script, informing the adaptation of the CSD matrix, and validating the decision to apply the Double Diamond methodology. This literature-informed framework ensured alignment with recent evidence in health informatics, user-centered digital health design, and interface evaluation—reinforcing its relevance to real-world EHR implementation.

### 2.4 Generative AI disclosure

In preparing this manuscript, the authors used ChatGPT (28) as a continuous support tool for translation and language refinement, aiming to improve clarity, consistency, and adherence to academic tone. As non-native English speakers, the authors employed this language model to help ensure fluency and precision throughout the text. Nevertheless, all content was thoroughly reviewed and edited by the authors, who take full responsibility for its scientific accuracy and integrity.

## 3 Results

This section presents the results of a real-world evaluation of the Afya iClinic digital ecosystem, combining service mapping and qualitative analysis of user experiences in mental health settings. The goal was to understand how different user profiles interact with the platform, to identify points of friction, usability barriers, and opportunities for system improvement. Key findings are organized thematically below, emphasizing the critical elements that guided the formulation of the proposed solutions.

### 3.1 Mapping the Afya iClinic digital ecosystem

The ecosystem analysis revealed the complexity of interactions between direct and indirect stakeholders, internal and external systems, and the regulatory frameworks that influence the use of the Afya iClinic platform. At its core, the EHR serves as the central hub, connecting healthcare professionals, patients, and clinic managers to essential services (4). The platform's success relies on its ability to facilitate seamless information flow across clinical and administrative domains.

The EHR centralizes patient health data, enabling longitudinal care management by professionals such as physicians, psychologists, dentists, and physiotherapists (1–4). It also interfaces with administrative tools critical for practice managers, allowing efficient coordination of operations and financial workflows (17). This core functionality grants users access to other essential modules such as appointment scheduling, electronic prescriptions, and telemedicine. Therefore, the platform must offer an intuitive and responsive interface capable of accommodating diverse user profiles, particularly during high-stakes clinical interactions (1–4).

The mapping exercise also underscored the need for platform personalization tailored to each stakeholder group. Direct users include healthcare professionals, patients, and managers. Professionals use the EHR for clinical and administrative purposes, patients engage through integrated portals to manage their data and schedule appointments, and managers rely on the platform to oversee operations, monitor billing, and generate financial reports (1–4).

In addition to these primary users, indirect stakeholders such as administrative staff, pharmacies, laboratories, and diagnostic centers play critical roles in the ecosystem. Secretaries manage scheduling and provide logistical support to both professionals and patients. Pharmacies interact with the system via electronic prescriptions, and diagnostic centers supply test results directly through the EHR interface (3, 4). This complex network of interactions reinforces the role of Afya iClinic as an essential tool for coordinated care and operational efficiency.

Finally, the ecosystem mapping highlighted the critical importance of regulatory compliance, particularly with regard to LGPD requirements (11, 12). Ensuring the security of sensitive patient data is essential to building trust and safeguarding ethical practice. As such, the platform must align with public health policies and be equipped to interface with governmental and regulatory systems, reinforcing its integrity as a secure and compliant digital health solution.

**Figure 2:**
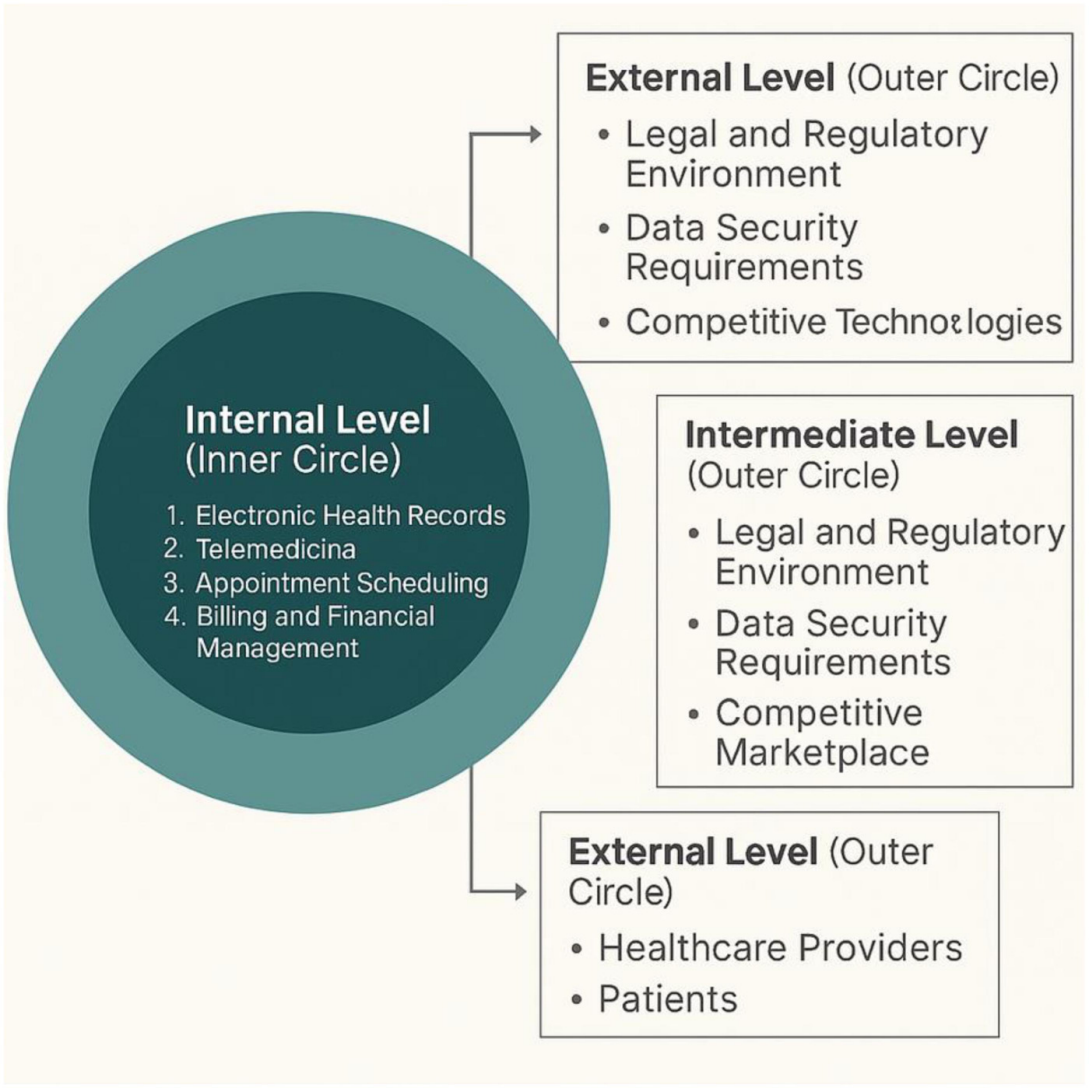
Ecosystem structure: core, intermediate, and external.

Figure 2 summarizes the digital ecosystem of the Afya iClinic platform, structured into three concentric layers that represent varying levels of proximity and function in relation to patient care. The Inner Layer features core users such as clinicians and patients directly engaging with the platform in a clinical context; the Intermediate Layer consists of administrative support roles, including electronic prescription processing, staff training, and medical marketing; and the Outer Layer represents external systems and integrations—such as laboratories, pharmacies, and insurance networks—that are critical to achieving interoperability. This layered model illustrates the ecosystem's complexity and interdependence, helping to optimize data flow across the healthcare continuum, and was further refined through the development of a service blueprint that details the interactions between user-facing components, backstage systems, and support infrastructure throughout the clinical workflow.

### 3.2 Interview analysis

Exploratory interviews conducted with healthcare professionals provided rich insights into the real-world use of the Afya iClinic platform, highlighting both its strengths and the challenges experienced by users. The study involved 21 participants, including psychiatrists, psychologists, general practitioners, and physiotherapists, ensuring a diverse representation in terms of clinical background, digital familiarity, and years of experience.

This heterogeneity was critical to capturing distinct experiences with the platform. Digital natives generally reported ease of navigation and a greater openness to automation features, while digital immigrants described a steeper learning curve and ongoing need for technical support to utilize the platform effectively.

Participants with over 15 years of experience tended to express more critical perspectives, drawing comparisons with other platforms and identifying limitations in the Afya iClinic interface—particularly regarding terminology and workflows misaligned with mental health practices. Conversely, younger professionals with less experience were more open to adopting the system, though they also recognized areas for interface improvement, especially concerning quick access to critical information.

The collected data revealed common patterns in user difficulties and guided the design of targeted solutions to improve usability and operational efficiency. While interviewees acknowledged that Afya iClinic supports the organization of appointments and data management, they also emphasized persistent usability barriers and limitations in interface personalization, particularly for mental health practitioners. Participants were selected intentionally to ensure representation across gender, age, experience, and digital fluency, as summarized in Table 2.

**Table 2:** Participant profiles by profession, experience, and digital familiarity.

Participant	Profession	Gender	Age range	Years of experience	Technology familiarity
1	Psychiatrist	Female	41–50	15+ years	Digital immigrant
2	Psychiatrist	Female	20–30	5 years	Digital native
3	Psychiatrist	Male	50+	15+ years	Digital immigrant
4	Psychiatrist	Male	31–40	10 years	Digital native
5	Psychiatrist	Female	41–50	15+ years	Digital immigrant
6	Psychiatrist	Female	20–30	5 years	Digital native
7	Psychologist	Male	31–40	10 years	Digital native
8	Psychiatrist	Female	41–50	15+ years	Digital immigrant
9	Physiotherapist	Female	20–30	5 years	Digital native
10	Psychologist	Male	50+	15+ years	Digital immigrant
11	Psychiatrist	Male	31–40	10 years	Digital native
12	Psychologist	Male	50+	15+ years	Digital immigrant
13	General practitioner	Male	31–40	10 years	Digital native
14	General practitioner	Female	41–50	15+ years	Digital immigrant
15	Psychiatrist	Female	20–30	5 years	Digital native
16	Psychiatrist	Male	50+	15+ years	Digital immigrant
17	Psychiatrist	Male	31–40	10 years	Digital native
18	Psychiatrist	Female	41–50	15+ years	Digital immigrant
19	Psychologist	Female	20–30	5 years	Digital native
20	Psychologist	Male	50+	15+ years	Digital immigrant
21	Psychiatrist	Female	41–50	15+ years	Digital immigrant

The user persona profiles developed in this study portray each participant's role as an archetype of platform interaction, capturing patterns of motivation, friction points, and usability expectations. These personas capture motivations, frustrations, and usability needs, providing a deep understanding of the challenges faced by healthcare professionals using Afya iClinic. Such insights were essential in shaping user-centered design solutions tailored to both clinical and administrative demands.

**Figure 3:**
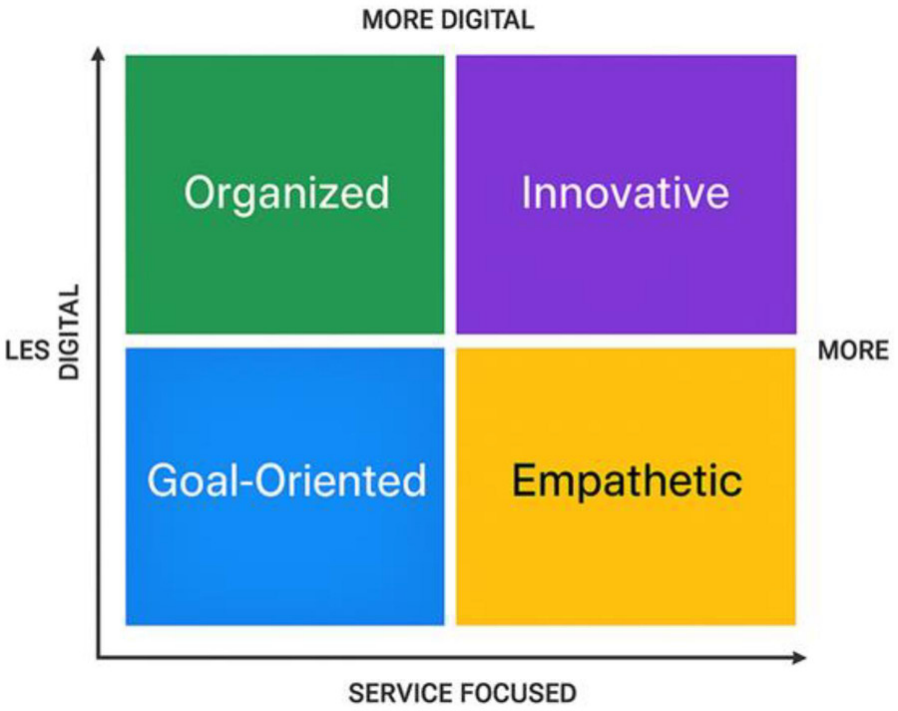
Classification of user profiles based on digital orientation and service focus.

The personas serve as narrative examples of representative user types and support the justification of interface improvements discussed throughout the study. Based on the qualitative characteristics of the participants presented in Table 2, a classification framework was developed to cluster user profiles according to their digital fluency and clinical interaction style. This typology is illustrated in Figure 3, which presents four dominant archetypes: Organized, Innovative, Goal-Oriented, and Empathetic.

### 3.3 Key issues and improvement opportunities

**Figure 4:**
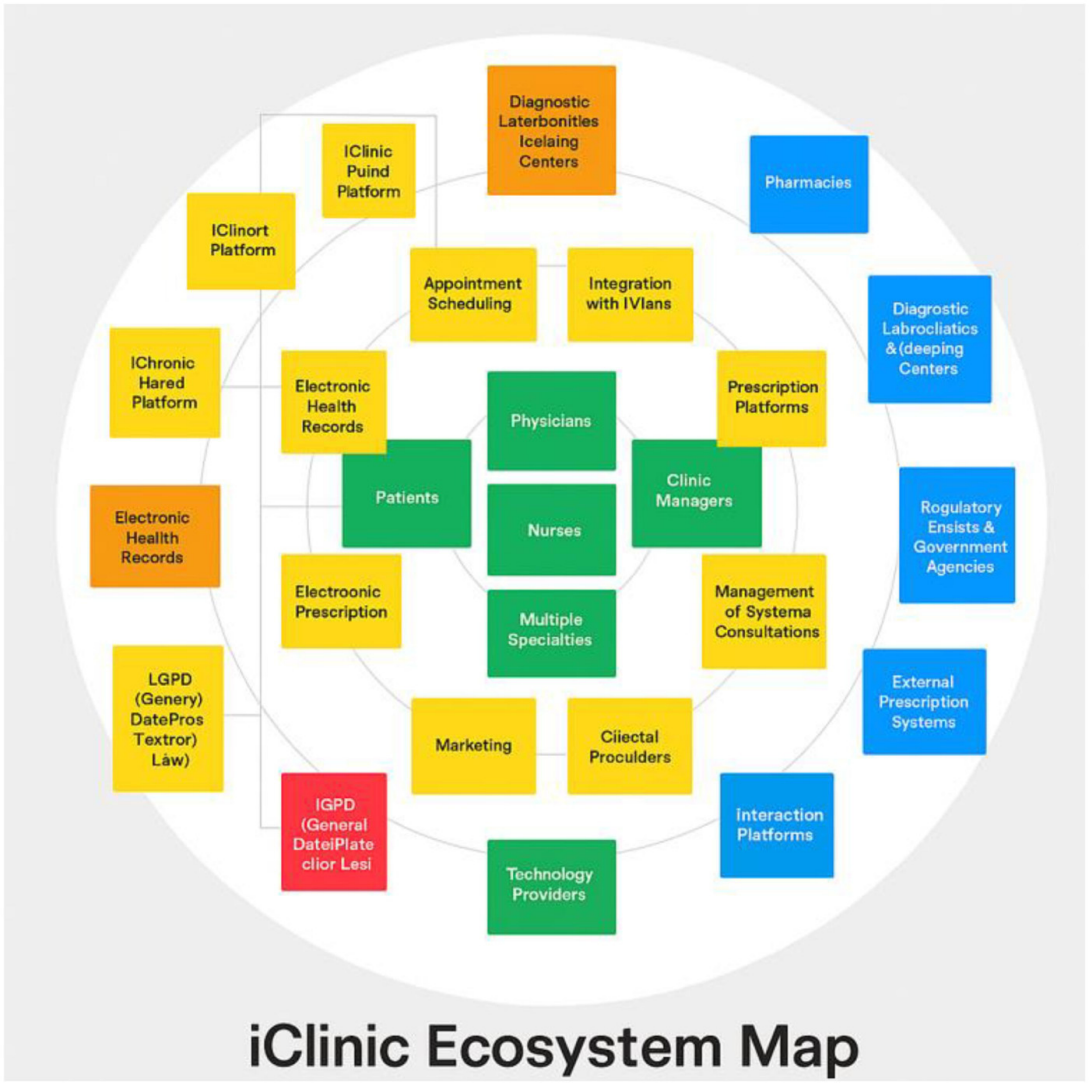
Afya iClinic ecosystem Map: stakeholder functional network.

The combined insights from ecosystem mapping and interview analysis revealed critical problems and corresponding opportunities for improvement, described in Figure 4. Primary challenges included interface inconsistencies and unclear error messages, which hindered system usability and increased reliance on technical support. In certain modules—particularly those focused on reporting and statistics—an overload of irrelevant information for some user profiles was observed. Psychologists, for example, noted that specific terms and functionalities were not aligned with mental health practice, indicating the need for specialty-based interface adjustments.

Another major issue was the lack of automation and shortcuts for repetitive tasks, such as quickly accessing patient records or generating prescriptions. This gap in operational efficiency was frequently cited as a source of user frustration. Additionally, inconsistent icons and unclear labels across interface sections impaired navigation and the ability to locate essential functions.

On a positive note, participants praised the platform's integration with pharmacies and laboratories, which helped streamline workflows and reduced manual processing. However, they also emphasized the need to expand these integrations to include other systems within the broader healthcare ecosystem, such as insurance providers and financial tools.

### 3.4 Clinical and administrative impact

Findings indicated both positive and negative impacts of the Afya iClinic platform on clinical and administrative routines. The system supports appointment organization and patient data management, particularly benefiting physicians and clinic managers. However, it still presents challenges for other user profiles—such as psychologists and administrative staff—due to limited contextual support and the absence of embedded help resources. These shortcomings hinder user autonomy and increase reliance on external assistance.

Limited customization of workflow structures was also identified as a constraint, particularly for professionals addressing specialized needs, such as those in mental health. Excessive dependence on technical support, combined with weak integration with external tools, underscores the necessity of redesigning both the interface and the information architecture.

### 3.5 Summary of results

Overall, the Afya iClinic platform operates as a robust digital health ecosystem but requires targeted refinements to enhance usability and efficiency across diverse user profiles. While it plays a pivotal role in clinical and administrative coordination, the system still faces usability limitations related to interface personalization, external integration, and user support. Collectively, these findings illustrate a multifaceted picture of the Afya iClinic platform's strengths and weaknesses. They serve as the empirical basis for the development of targeted, user-informed recommendations, which are presented in the next section.

### 3.6 Recommendations and design proposals

Based on the findings of this study—drawn from both user interviews and heuristic evaluations—this section presents a set of evidence-informed recommendations that aim to enhance usability and improve user satisfaction with the Afya iClinic platform. It is important to note that this analysis was conducted independently and not commissioned by Afya iClinic or its affiliates. Therefore, the proposals presented here serve as academic and exploratory contributions, with the intent of fostering future dialogue and improvements in digital health platforms.

A critical recommendation is the standardization of the interface across key modules such as the appointment scheduler and the EHR. Inconsistencies in layout and terminology were found to contribute to user confusion and cognitive load, particularly among professionals who engage intensively with the platform. Enhancing design uniformity is essential for providing a more fluid and intuitive experience (1–4).

**Figure 5:**
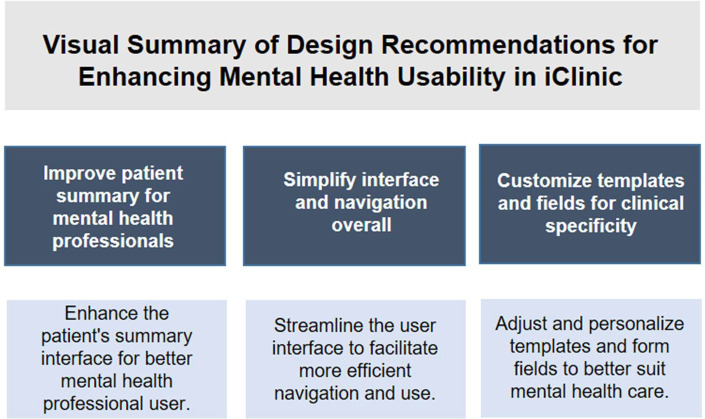
User quotes clustered by theme: friction points in interface and workflow.

Another priority is the implementation of real-time validation and contextual guidance during data entry. These features could help reduce input errors and increase the accuracy of recorded information, thereby minimizing reliance on technical support for minor issues. Likewise, automating repetitive tasks and enabling user-defined shortcuts would streamline workflows, allowing professionals to customize their interface according to their specific clinical routines (2–4). The lack of such features was frequently identified as a barrier to operational efficiency, particularly among users managing high patient volumes. Figure 5 groups representative user quotes into four thematic clusters, each illustrating recurring pain points across user profiles.

Context-sensitive help integrated directly into the platform's interface is also strongly recommended. Interactive tutorials and explanatory videos could provide immediate support and facilitate autonomous problem-solving, especially for users with limited digital proficiency (3). Similarly, the current handling of error messages—described by users as vague and unhelpful—should be revised. Clear, informative alerts with actionable suggestions would significantly reduce user frustration and prevent workflow disruptions.

**Figure 6:**
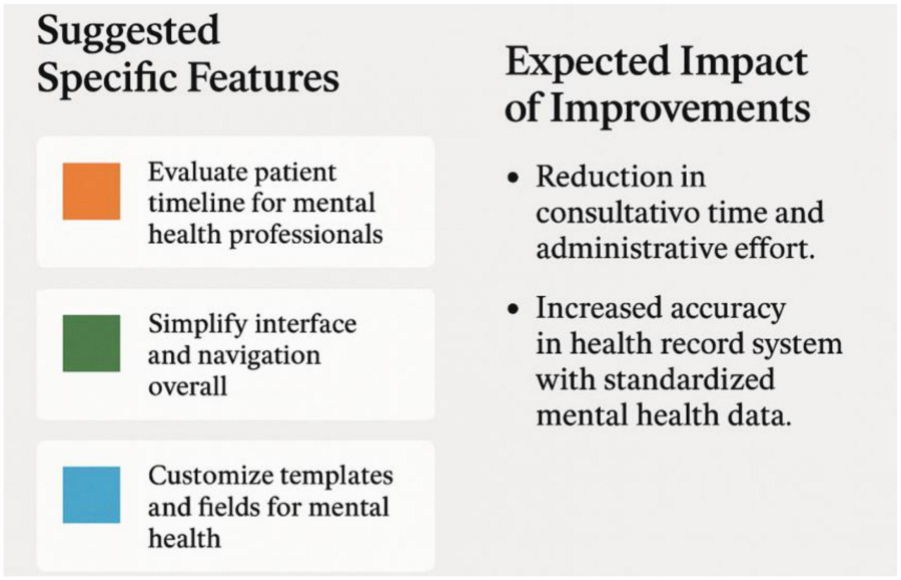
Visual summary of design recommendations for enhancing mental health usability in afya iClinic.

These recommendations are especially pertinent to the needs of mental health professionals, such as psychologists, whose clinical workflows and documentation practices differ from those of other medical fields. A tailored implementation strategy, grounded in iterative testing and user feedback, should validate each enhancement before broader application. Ensuring alignment between new functionalities and actual user expectations is critical to achieving a more integrated and user-friendly platform (1–4). A summary of design-focused recommendations is visually presented in Figure 6.

While not commissioned by Afya iClinic, this study suggests that embracing these recommendations could substantially elevate the overall quality of the platform. Improvements in usability, customization, and integration would enhance not only patient care but also the internal operations of clinics and private practices. These refinements could ultimately enhance user trust and contribute to the platform's evolution as a more integrated and user-responsive digital health solution.

The application of these recommendations must account for the diversity of user profiles identified in this study. Figure 3 presents a classification matrix based on two axes: digital orientation and care delivery focus. This typology supports the personalization of platform improvements to better serve both highly digital users and those requiring greater assistance.

**Figure 7:**
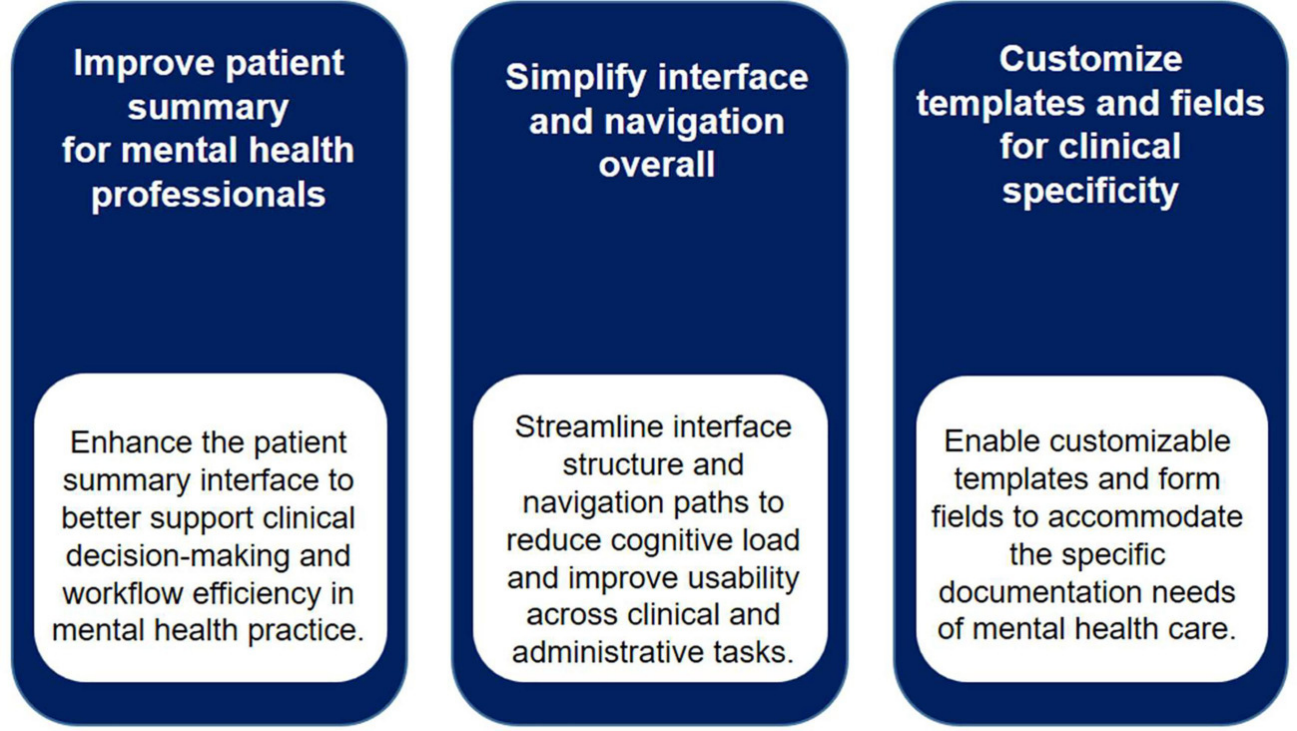
Visual summary of design recommendations for enhancing mental health usability in afya iClinic.

The matrix includes four user archetypes—Organized, Innovative, Goal-Oriented, and Empathetic—each with distinct preferences and usability needs. Recognizing this spectrum of user characteristics enables more targeted and effective enhancements. For example, recommendations such as interface standardization, real-time validation, and contextual help can be selectively applied to address the unique expectations of each user group, resulting in a more coherent and efficient experience for all platform stakeholders. Figure 7 outlines the specific features proposed and their projected impacts on workflow efficiency and data quality.

### 3.7 Design translation

**Figure 8:**
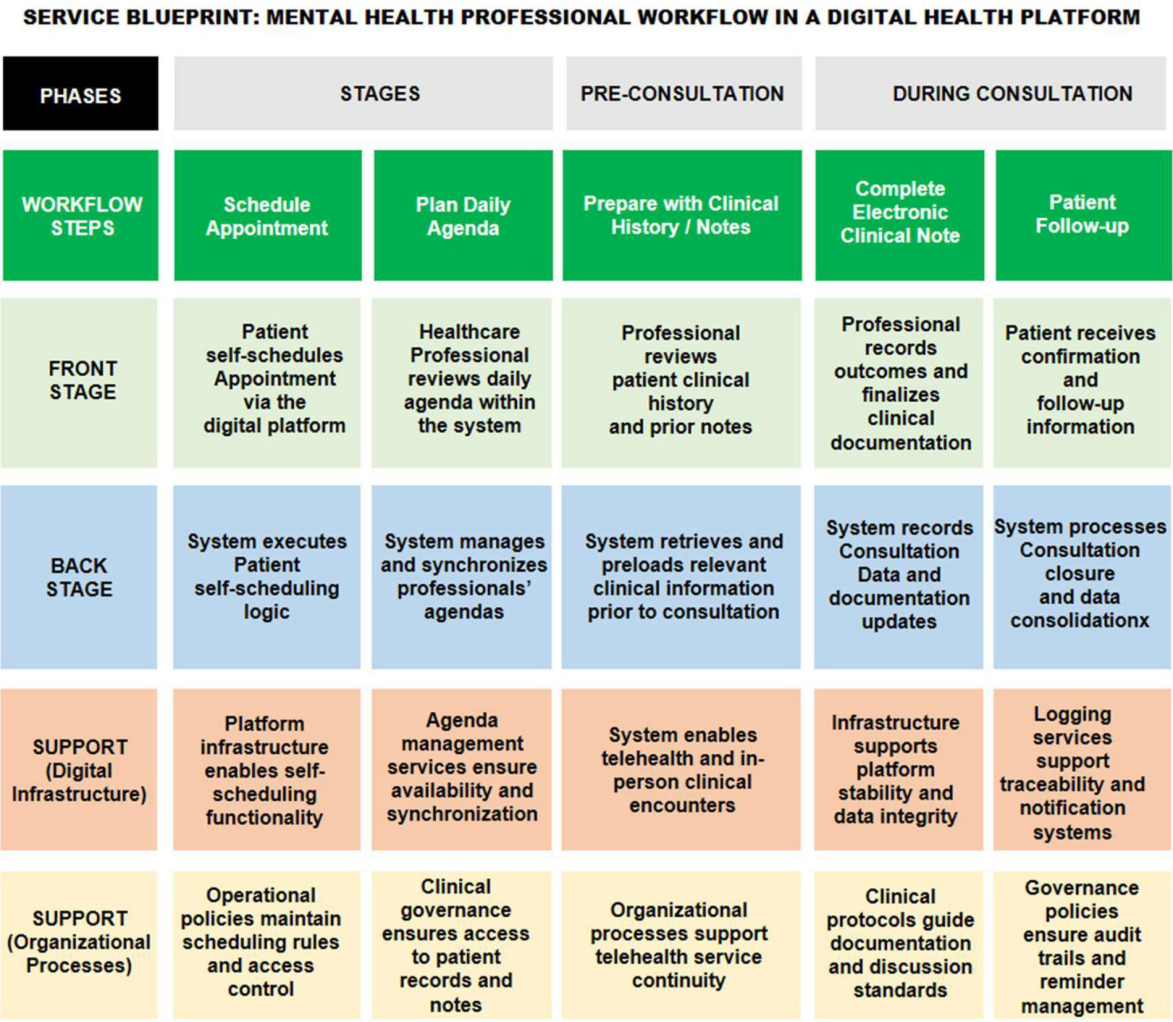
Service Blueprint: Consultation Journey in Afya iClinic from Onboarding to Follow-up.

The blueprinting and prototyping phases were grounded in the thematic insights and user personas developed earlier in the study. The service blueprint (Figure 8) provides a systemic representation of the consultation journey, detailing not only user-facing interactions but also backstage processes and support infrastructure. This visualization clarifies how different actors, technologies, and workflows intersect throughout the clinical encounter.

**Figure 9:**
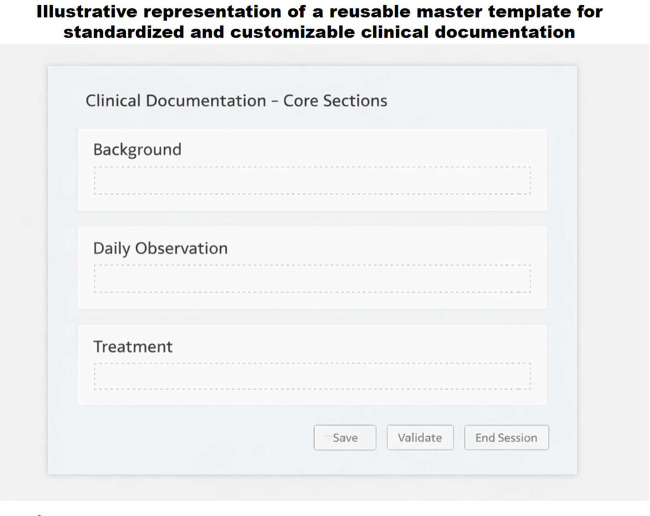
Prototype Interface Mockup Reflecting Simplified Documentation Flow.

Building on this systemic understanding, a conceptual prototype interface was developed (Figure 9) to represent a simplified and customizable documentation flow grounded in the usability issues identified in earlier phases. The interface was designed to minimize visual clutter, enable user-specific template customization, and incorporate real-time data validation features. These elements were directly informed by the usability issues identified in the Discover and Define phases.

The resulting prototype embodies the study's design-driven recommendations and serves as a foundational model for usability improvements and future iterative testing. It operationalizes key insights into actionable interface solutions and establishes a tangible bridge between user experience research and EHR implementation practice.

The following section discusses these findings in light of broader design implications and system-level considerations.

## 4 Discussion

The discussion of findings reveals that while Afya iClinic provides valuable features for both clinical and administrative use, it still faces significant barriers in terms of usability, system integration, and support for diverse user profiles. Interviews with mental health professionals highlighted points of friction and platform-specific limitations, as well as opportunities to improve efficiency and better align with the evolving needs of the healthcare sector. These insights were further supported by heuristic evaluation, which provided a foundation of best practices in user interface design to guide the proposed recommendations and highlighted how incremental design refinements—grounded in real user behavior—can drive meaningful improvements in EHR implementation strategies.

One of the most critical issues raised was related to navigation and user flow. While the interface was initially perceived as intuitive, users reported that transitioning between sections was often slow. This lack of fluidity was particularly problematic during clinical appointments, where quick access to essential information is crucial. Heuristic analysis confirmed these perceptions, showing that inconsistent design patterns increase cognitive load and diminish user experience. To address this, customized shortcuts and layout improvements were suggested to streamline transitions between EHR, scheduling, and reporting modules.

Another major limitation was the lack of flexibility in session templates, which affected mental health professionals such as psychologists. The platform's generalized structure fails to accommodate the specific documentation and workflow needs of these users, requiring time-consuming manual adjustments. Heuristic assessment identified this inflexibility as a misalignment with real-world practice, suggesting that the platform should allow customization of fields and processes based on specialty. This type of personalization would make the system more relevant and efficient across a wider range of clinical contexts.

Interviewees noted that although Afya iClinic includes basic communication tools such as email and SMS reminders, the absence of integration with commonly used applications like WhatsApp limits its alignment with real-world communication workflows. Centralizing these interactions within the platform would facilitate communication and save time, reducing the need to toggle between multiple tools. Heuristic principles related to flexibility and efficiency reinforce the value of such integrations, which would streamline patient engagement and provider workflows.

Users further reported difficulties generating and sharing clinical reports. The platform's limited formatting and export options hinder interprofessional collaboration and often result in manual workarounds and redundancies. These constraints conflict with the heuristic principle of error prevention and the design guideline that systems should support recognition rather than recall, highlighting the importance of aligning digital tool functionalities with evidence-based interface design principles to support interdisciplinary collaboration. Improving these functionalities would enable professionals to adapt reports to various clinical scenarios, supporting more collaborative and integrated care delivery.

These usability limitations, when viewed through the lens of the service blueprint, reveal structural inefficiencies that go beyond interface design. The blueprint helped visualize how gaps in backstage support and workflow logic contribute to user frustration. Moreover, the development of interface prototypes allowed the team to explore potential solutions in practice, highlighting the transformative role of design as both analytical and generative in the healthcare context.

On the administrative side, participants found the scheduling and payment management systems useful but noted areas needing improvement. A lack of flexibility in configuring alternative appointment times and making quick adjustments hindered agenda management. Moreover, limited integration with payment platforms created obstacles in billing workflows, such as generating invoices or processing transactions. Addressing these shortcomings could simplify administrative processes and reduce time spent on routine tasks.

Technical support and update management also emerged as important concerns. While overall support was rated positively, users reported delays in issue resolution and disruptions caused by unannounced platform updates. These interruptions compromised operational continuity and diminished user confidence. Interviewees emphasized the need for more transparent communication regarding updates and a more responsive support infrastructure to enhance the reliability of the user experience.

Lastly, the lack of embedded self-learning resources—such as tutorials and explanatory videos—was seen as a limiting factor. Without contextual help integrated directly into the platform, users with lower digital literacy levels must rely heavily on technical assistance. A built-in knowledge base would improve autonomy and enable more efficient problem-solving, especially for those new to digital health systems.

Taken together, these findings reaffirm that Afya iClinic has the potential to offer a more streamlined and integrated experience but must overcome key challenges. Users consistently called for enhanced usability, greater feature flexibility, and stronger integration with third-party tools. The platform must also evolve to meet the particular demands of mental health professionals by offering specialty-specific templates and adaptable workflows. Improved technical support and effective help resources are essential for ensuring a smoother, less frustrating user journey.

The results discussed in this section lay a strong foundation for the development of targeted recommendations and point to practical directions for platform refinement. Validation through usability testing and prototyping will be critical to ensure that any implemented changes align with user expectations. Successfully applying these enhancements would help Afya iClinic strengthen its role as a comprehensive platform for managing both clinical practice and administrative tasks, maximizing its value across diverse healthcare environments.

From a design research perspective, this study illustrates how the integration of systemic mapping, persona modeling, and heuristic evaluation can form a replicable methodological framework for improving EHR usability in diverse healthcare environments. It reinforces the potential of service design tools not only for identifying pain points, but also for articulating feasible, context-aware improvements that respect the complexity of healthcare systems. The iterative nature of this process exemplifies how design can mediate between user needs and system constraints in real-world digital platforms.

These reflections underscore the symbolic and functional potential of digital health platforms like Afya iClinic—not only as technological tools, but as dynamic interfaces that actively shape clinical reasoning, user satisfaction, and the sustainability of healthcare operations. The conclusions that follow consolidate these insights into a broader vision for platform evolution, methodological advancement in design research, and future applications in digital health innovation. Taken together, these findings suggest that design-driven, user-centered refinements can operate as managerial levers that potentiate value creation and resilience at the organizational level, extending beyond the interface to the governance of digital health innovation.

## 5 Conclusions

This study evaluates the Afya iClinic platform within the context of mental healthcare, identifying implementation challenges and proposing design-based recommendations to enhance user experience and operational efficiency. Through qualitative interviews, heuristic evaluation, and digital ecosystem mapping, the research uncovered how various user profiles—including psychologists, physicians, patients, and managers—interact with the platform and what their primary needs and friction points are.

The findings demonstrated that while Afya iClinic offers useful functionalities such as scheduling, EHRs, and basic financial service integration, key limitations negatively impact overall system efficiency and usability. Among the most significant challenges was the lack of interface customization, particularly for mental health professionals like psychologists, who require greater flexibility to tailor templates and records to specific clinical needs. Additionally, navigation was reported to be slow and click-intensive, impairing workflow fluency during consultations and increasing the cognitive burden on users. Limited integration with popular communication tools like WhatsApp and more robust financial systems further hindered administrative efficiency and contributed to duplicative work processes.

Another critical issue was related to error messaging and unannounced updates. Interviewees reported workflow interruptions and growing user frustration due to sudden changes and the absence of clear guidance for resolving technical issues. The excessive reliance on technical support, stemming from the lack of embedded self-learning resources and a comprehensive knowledge base, underscored the need for a more autonomy-driven approach to user interaction.

Based on these findings, targeted recommendations were proposed to improve the platform. These included interface personalization for different medical specialties, especially mental health; streamlined navigation supported by shortcuts and automation; and expanded integration with both communication and financial tools. Enhancements in error communication and update transparency were also emphasized, along with the implementation of contextual help resources such as tutorials and explanatory videos to reduce support dependency.

Beyond its contextual findings, this study contributes to the field of design research by demonstrating how user-centered methodologies—such as service blueprints, persona modeling, and heuristic analysis—can be synthesized to generate actionable outcomes in complex digital ecosystems. The methodological approach adopted may serve as a replicable and adaptable model for similar evaluations in other healthcare platforms and digitally mediated service environments.

While this study offers important insights into the usability and workflow dynamics of the Afya iClinic platform, several limitations must be acknowledged. First, the qualitative data were collected from a relatively small and specialized sample of mental health professionals, which may limit the generalizability of the findings across broader healthcare contexts. Second, the absence of a practical implementation phase—such as real-world testing of proposed prototypes—restricts the validation of the recommendations in applied settings. Third, the study focused primarily on user experience and interface design and did not assess technical performance indicators or clinical outcomes.

Future research should expand the sample to include other healthcare specialties and organizational contexts, such as general medicine, physiotherapy, or dental practices. Additionally, the integration of usability testing with functional pilot implementations will be essential to validate the proposed design changes and measure their impact on clinical efficiency, user satisfaction, and administrative workflows. Exploring the integration of artificial intelligence, personalized analytics, and real-time feedback mechanisms may also offer promising directions for platform innovation and adaptation to increasingly complex digital health ecosystems.

In summary, this study offers a deeper understanding of the challenges and opportunities associated with using Afya iClinic in the mental health domain. The analyses conducted suggest that with targeted adjustments and design innovations, the platform has strong potential to deliver a more effective and satisfying experience for both clinical and administrative users. Adoption of the recommendations presented here could help Afya iClinic deliver greater value to its users and solidify its role as an essential tool for managing modern clinics and healthcare practices. These insights may also benefit other digital health platforms, supporting continuous improvement in user experience and system design, and contributing to the advancement of digital health innovation in middle-income healthcare settings.

Ultimately, this research not only improves our understanding of a specific platform but also underscores the role of design as a transformative practice in digital health. By integrating user voices, system mapping, and prototyping into a coherent process, the study offers a pathway for developing more human-centered, efficient, and ethically grounded health technologies. Its implications extend to the broader discourse on digital infrastructure, clinician well-being, and the symbolic and systemic dimensions of care delivery in the 21st-century healthcare landscape.

## Data Availability

The original contributions presented in the study are included in the article/Supplementary Material, further inquiries can be directed to the corresponding author.
